# *Escherichia coli* BW25113 Competent Cells Prepared Using a Simple Chemical Method Have Unmatched Transformation and Cloning Efficiencies

**DOI:** 10.3389/fmicb.2022.838698

**Published:** 2022-03-24

**Authors:** Yuqing Yang, Qiaoli Yu, Min Wang, Rui Zhao, Huaiwei Liu, Luying Xun, Yongzhen Xia

**Affiliations:** ^1^State Key Laboratory of Microbial Technology, Shandong University, Qingdao, China; ^2^Institute of Marine Science and Technology, Shandong University, Qingdao, China; ^3^School of Molecular Biosciences, Washington State University, Pullman, WA, United States

**Keywords:** transformation, competent cells, *Escherichia coli*, recA, cloning, recombination

## Abstract

*Escherichia coli recA*^−^ strains are usually used for cloning to prevent insert instability *via* RecA-dependent recombination. Here, we report that *E. coli* BW25113 (*recA*^+^) competent cells prepared by using a previously reported transformation and storage solution (TSS) had 100-fold or higher transformation efficiency than the commonly used *E. coli* cloning strains, including XL1-Blue MRF’. The cloning success rates with *E. coli* BW25113 were 440 to 1,267-fold higher than those with *E. coli* XL1-Blue MRF’ when several inserts were assembled into four vectors by using a simple DNA assembly method. The difference was in part due to RecA, as the *recA* deletion in *E. coli* BW25113 reduced the transformation efficiency by 16 folds and cloning success rate by about 10 folds. However, the transformation efficiency and the cloning success rate of the *recA* deletion mutant of *E. coli* BW25113 are still 12- and >48-fold higher than those of *E. coli* XL1-Blue MRF’, which is a commonly used cloning strain. The cloned inserts with different lengths of homologous sequences were assembled into four vectors and transformed into *E. coli* BW25113, and they were stably maintained in BW25113. Thus, we recommend using *E. coli* BW25113 for efficient cloning and DNA assembly.

## Introduction

DNA cloning is a common technique used in biological research, such as constructing CRISPR vectors and plasmids encoding metabolic pathways (Ellis et al., 2011; Sanjana et al., 2014). The conventional method relies on DNA digestion by restriction enzymes and re-ligation by T4 DNA ligase (Cohen et al., 1973; Smolke, 2009). Recently, seamless cloning methods have gained adaptation to meet the requirements of higher efficiency, fidelity, and modularity (Kosuri and Church, 2014). DNA fragments with homology ends or ends that can be recognized by a phage recombinase are generated and assembled either *in vivo* or *in vitro* (Fu et al., 2008; Chao et al., 2015). For *in vivo* methods, these DNA fragments are joined in host cells, such as *Saccharomyces cerevisiae* or *Escherichia coli*. When *E. coli* is used, it usually overexpresses a bacteriophage recombination system to join DNA fragments (House et al., 2004; Wang et al., 2016). For *in vitro* methods, DNA fragments are treated with enzymes to join DNA fragments. The *in vitro* methods are widely used for routine cloning, and *in vivo* methods that often require longer homologous ends are more useful in the assembly of large plasmids (Zhu et al., 2007; Jeong et al., 2012; Zhang et al., 2012). We have developed a “T5 exonuclease DNA assembly” (TEDA) method that combines *in vitro* digestion by 5′-3′ exonuclease with the rest recombination inside *E. coli* to join homology ends with high efficiency for routine cloning at low cost, and the method works better with chemically prepared competent cells than with electroporation (Xia et al., 2019).

*E. coli* K-12 derivatives with a defective *recA* (*recA1*) that is defective in recombination are often used for cloning (Bullock, 1987; Anton and Raleigh, 2016). DH5α is engineered to enable blue white screening for the β-galactosidase (LacZ) activity (Hanahan, 1983). XL1-Blue MRF’ with all restriction systems being removed is often used to clone methylated DNA especially from eukaryotes (Bullock, 1987). JM109 is considered as a better choice to clone repetitive DNA (Yanischperron et al., 1985; Metcalf and Wanner, 1993). Stbl2 is a derivative of JM109 (Trinh et al., 1994), and Stbl3 is a hybrid strain of *E. coli* K-12 and *E. coli* B; both are developed to clone direct repeats, retroviral sequences, and tandem array genes (Al-Allaf et al., 2013). Derivatives from other *E. coli* strains are also used. Mach1, derived from *E. coli* W, is a fast-growing *recA*^−^ strain (Green and Sambrook, 2018). The commonly used *E. coli recA*^+^ strain is BL21(DE3) for the expression of recombinant proteins. Although BL21(DE3) is *recA*^+^, its transformation efficiency (TE) is much lower than commonly used cloning strains derived from K-12 (Chan et al., 2013). Clearly, *E. coli* K-12 *recA*^−^ strains are the common cloning *E. coli* strains.

The common cloning *E. coli* strains often contain additional mutations to benefit cloning. The mutation of the *hsdR* or *hsdS* gene prevents the unmethylated DNA from being degraded (Sain and Murray, 1980). The *endA* mutation avoids random degradation of DNA by endonuclease I after cell lysis, improving both the quantity and quality of plasmid extraction (Lin, 1992; Altermark et al., 2007). DeoR regulates cells’ ability to continuously synthesize deoxyribose, whose mutation increases cells’ ability to absorb large plasmid DNA (Hanahan et al., 1991). The *galE* mutation reduces the interference from lipopolysaccharide for DNA uptake (van Die et al., 1984). FhuA serves as the receptor for phages T5, T1, φ80, and UC-1 (Hantke and Braun, 1975, 1978; Carmel and Coulton, 1991), and its deletion protects cells from phages infection especially during library construction (Bonhivers et al., 1998). The mutation of these genes is commonly employed for constructing cloning strains.

The *recA* gene in almost all *E. coli* cloning strains is inactivated to increase plasmid stability (Clark, 1973). RecA is the core component of homologous recombination (Maraboeuf et al., 1995; Kowalczykowski, 2000; Morimatsu and Kowalczykowski, 2014), and it catalyzes intermolecular recombination to generate dimers that lead to plasmid loss without selection pressure (Summers et al., 2010; Standley et al., 2019). Low copy number plasmid is relatively stable in *recA*^+^ strains, but high copy number plasmids easily form multimers and generate plasmid-free cells without selection (Herman-Antosiewicz and Wegrzyn, 1999). With selection pressure, the subpopulation with dimers in *recA*^+^ cells is confined due to the reduced growth for cells with plasmid dimers (Summers et al., 2010). Other concerns related to RecA are homologous recombination events within a plasmid or between a plasmid and the chromosome (Sambrook et al., 2017). Although these are valid concerns, direct evidence on their effects on cloning has not been reported.

We report here that *E. coli* BW25113, a K-12 derivative with a *recA*^+^ and *hsdR^−^* genotype and the parent of the Keio collection of single-gene knockouts (Baba et al., 2006), had >100-fold higher TE than the commonly used cloning *E. coli* hosts when intact pBluescript SK- (pSK-) was chemically transformed by using a single step method of the transformation and storage solution (TSS). The cloning success rates with BW25113 were 440 to 1,267-fold higher than those with XL1-Blue MRF’ when various inserts were assembled into 4 vectors by the TEDA method. The cloned inserts with different lengths of homologous sequences were stable in BW25113. Our results promote the reconsideration of using the *recA*^+^ strain *E. coli* BW25113 for DNA assembly in synthetic biology with significantly increased success rates.

## Materials and Methods

### Strains and Plasmids

The strains used in this study were listed in Supplementary Table S1. All mutants in this research were obtained according to the Wanner’s method (Datsenko and Wanner, 2000). All strains were cultured at 37°C in Luria-Bertani (LB) medium with appropriate antibiotics. Ampicillin, spectinomycin, gentamycin (Gm), chloramphenicol, and kanamycin (Kan) were used at 100, 50, 10, 25, and 50 μg/ml, respectively.

### Enzymes and Reagents

Phusion DNA polymerase (Thermo Fisher, United States) was used to amply DNA fragments. PrimeSTAR GXL DNA Polymerase (Takara, Japan) was used to amply DNA fragments whose length are larger than 10 kb. Trans 5 K and 1 kb DNA markers (TransGen Biotech, Beijing) were used to measure the size of DNA fragments in agarose gel electrophoresis. Gel extraction kit (Omega, United States), Plasmid extraction mini Kit (Omega, United States), and TIANamp Bacteria DNA Kit (TianGen, China) were used to purify DNA fragments. All oligos were synthesized by Beijing Genomics Institute. Magnesium chloride, manganese chloride, PEG8000, PEG3350, and dimethylsulfoxide (DMSO) were purchased from Sigma-Aldrich (US), and the rest reagents were purchased from Sangon Biotech (China).

### Procedures for Competent Cells Preparation and Transformation

To prepare competent cells, a fresh single colony was normally inoculated into 4-mL LB medium and incubated at 37°C overnight. Then, 1% culture was transferred to 50 ml fresh LB medium and cultured at 37°C until OD_600_ around 0.5 to prepare competent cells for the TSS method and at 20°C to prepare competent cells for the Hanahan and Inoue methods. The growth was stopped by incubating on ice for 10 min. Cells were harvested by centrifuging at 4°C, 4000 *g* × 10 min, for the preparation of competent cells with different methods.

The TSS method was modified from two references (Chung et al., 1989; Parrish et al., 2004). Briefly, the harvested cells were resuspended in 1 ml of the TSS buffer (LB-HCl pH = 6.1, 10% PEG3350, 5% DMSO, 10% Glycerol, 10 mM MgSO_4_, and 10 mM MgCl_2_), and chilled on ice for 10 min. This mixture was aliquoted into 30 μl per tube. The preparation steps of competent cells were mainly adopted from the study of Chung et al. (1989). In the transformation step, 5 μl of 5 × KCM (0.5 M KCl, 150 mM CaCl_2_, and 250 mM MgCl_2_) was mixed with DNA and ddH_2_O to total 25 μl mixture. The mixture was gently mixed with 25 μl competent cells and incubated on ice for 30 min. A total of 250 μl fresh LB medium was added for cell recovery at 37°C for 1 h. Recovered cultures were spread into the LB plate with the indicated antibiotic. KCM was reported by Parrish et al. (2004).

For the Inoue method (Green and Sambrook, 2020), the harvested cells were washed with 20 ml of the Inoue buffer (55 mM MnCl_2_, 15 mM CaCl_2_, 250 mM KCl, and 10 mM PIPES pH = 6.7) and finally resuspended in 1 ml of the Inoue buffer. Then, 75 μl DMSO was added, and the mixture was incubated on ice for 10 min. The cell mixture was aliquoted into 100 μl/tube and could be stored at −80°C.

For the Hanahan method (Green and Sambrook, 2018), the harvested cells were washed with the CCMB80 buffer (80 mM CaCl_2_, 20 mM MnCl_2_, 10 mM MgCl_2_, 10% Glycerol, and 10 mM Potassium acetate buffer pH = 7.0) and finally resuspended in 1 ml CCMB80 buffer. The cell mixture was aliquoted into 100 μl/tube and could be stored at −80°C before use.

The transformation procedures used with the Inoue and the Hanahan method were similar. A 100 μl of competent cells was thawed on ice and mixed with DNA solution. The mixture was incubated on ice for 30 min before heat shock at 42°C for 90 s. After heat shock, 900 μl of fresh LB was added for cell recovery at 37°C for 1 h. Recovered cultures were spread into the LB plate with the indicated antibiotic.

All solutions were autoclaved and used under an ice-cold condition. All operations were performed on ice. The cells were gently resuspended in different buffers. The supernatant after centrifugation was cleanly and quickly pipetted off. The prepared competent cells could be stored at −80°C before use.

The TEs were tested by transforming 0.2 ng intact pBluescript SK- into the testing competent cells. The number of recovery colonies was counted and normalized to 1 μg of the plasmid DNA.

### The Preparation of Vectors and Inserts for DNA Assembly

The plasmids and primers used in this study are listed in Supplementary Tables S1 and S2, respectively. The TEDA method was used for plasmid constructions (Xia et al., 2019). Competent cells of defined strains prepared with different methods were used to transform either intact plasmids or DNA cloning mixtures.

Plasmids with artificial homologous characteristics were generated to check for their stability in *E. coli recA*^+^ strains. To construct plasmids with homology to *E. coli* genome, a 3,000-bp fragment was amplified from BW25113 genomic DNA near the *rapA* gene. The DNA fragment was assembled into plasmids with different replicons.

Direct repeats (DR) were introduced into plasmids through DNA assembly of four DNA fragments. The 5′ end of Kan-resistant gene (5’Kan), the intact Gm resistant gene (Gm), and the 3′ end of Kan-resistant gene (3’Kan) were assembled in pCL1920. Gm was inserted into the open reading frame (ORF) of Kan-resistant gene between 135 and 136 bp. A defined length of 300 bp from 136 to 435 bp of Kan ORF was amplified and inserted before Gm, so that a homologous recombination with the DRs will restore Kan resistance.

Inserts of short and long highly tandem repeats were introduced into plasmids through DNA assembly. For short repeats, 41-bp Tac promoter repeat (TR41) was used. The Tac promoter repeats have been assembled together to promote eGFP expression in a list of plasmids (Li et al., 2012). Fragments harboring 1, 4, 6, or 8 TR41 with an eGFP gene were amplified from corresponding plasmids and cloned into pCL1920. For medium-sized repeats, 300-bp from the C-terminal of eGFP was amplified and inserted after the gene in pCL1920::Pkat-eGFP to generate pCL1920::2TR300 with two repeats and pCL1920::3TR300 with three repeats. The interspace between the three TR regions was 104 and 67 bp. For long repeats, a defined 1-kb fragment containing Pkat-eGFP (TR1000) from pCL1920::Pkat-eGFP was amplified and inserted next to the TR1000 region to produce pCL1920::2TR1000 with two tandem repeats and pCL1920::3TR1000 with three tandem repeats. The interspace between the three tandem repeats regions was 65 and 67 bp. For testing, the inserts harboring tandem repeats were amplified and cloned back into the original pCL1920 plasmid by transforming indicated competent cells.

The TEDA method was used to assemble DNA fragments into various vectors (Xia et al., 2019). The *egfp* gene under the control of Pkat promoter (Pkat-eGFP) with 20-bp homology ends to SmaI-pSK was used, as previously described (Xia et al., 2019).

### The Screening for Positive Colonies and the Stability of Plasmid in Cells

The phenotype was used for initial screening to obtain the positive cloning rates (positive colonies/total colonies). Colonies producing eGFP were green. Further, 20 colonies were checked by using colony PCR. Ten plasmids from the positive colonies were extracted and checked through agarose gel electrophoresis and DNA sequencing (TsingKe BioTech, China).

The stability of plasmids with homology characteristics was initially checked for positive cloning rates. The stability of these plasmids was then tested. Five colonies were randomly picked and cultured in LB medium with appropriate antibiotics. The cultures were continuously transferred into fresh LB medium once reached the stationary phase. After three transfers, the plasmids were extracted and checked by using agarose gel electrophoresis and DNA sequencing.

## Results

### BW25113 Has a Significantly Higher TE Than Commonly Used *E. coli* Strains

**Figure 1:**
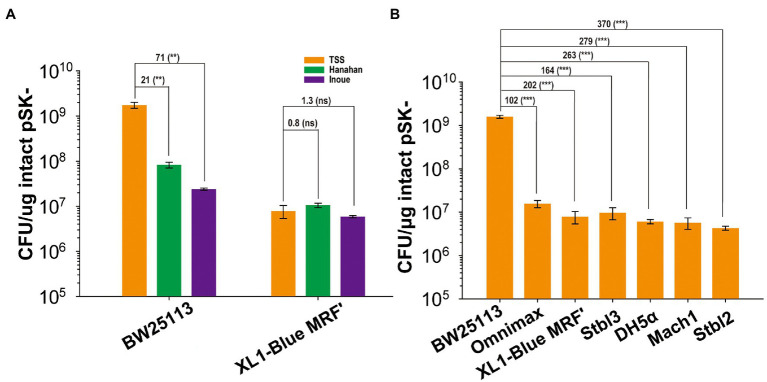
The TEs of *E. coli* strains. **(A)** The TEs of competent cells prepared with three different methods for two *E. coli* strains. **(B)** The TEs of competent cells prepared with TSS for seven *E. coli* strains. BW25113, Omnimax, XL1-Blue MRF’, Stbl3, DH5α, Mach1, and Stbl2. Data are averages of three samples with standard deviations (error bars). Unpaired t-tests were performed (** = *p* < 0.01; *** = *p* < 0.001; and “ns” = *p* > 0.05). “ns” means “not significant.” The numbers before (***) represent the difference by folds.

The *E. coli* BW25113 cells were made into competent cells by using three commonly used chemical methods: the Hanahan, Inoue, and TSS methods (Chung et al., 1989; Parrish et al., 2004; Green and Sambrook, 2018, 2020). The TSS method gave the highest TE when pSK- was transformed, and its TE was 21- and 71-fold higher than those of the Hanahan and Inoue methods, respectively (Figure 1A). When the *E. coli* XL1-Blue MRF’ competent cells were prepared by the three methods, the TEs were similar with overlapping error bars (Figure 1A). The TE values of *E. coli* BW25113 were higher than those of *E. coli* XL1-Blue MRF’ by 217, 8.1, and 4.6 folds when prepared using the TSS, Hanahan, and Inoue methods, respectively (Figure 1A). The TSS method is a simple method, in which the harvested *E. coli* cells are resuspended in TSS, stored at −80°C, and used for transformation (Chung et al., 1989; Parrish et al., 2004). The TSS method was then selected to prepare competent cells of BW25113 and several commonly used cloning *E. coli* strains. The TE of BW25113 was 102-, 202-, 164-, 263-, 279-, and 370-fold higher than that of Omnimax, XL1-Blue MRF’, Stbl3, DH5α, Mach1, and Stbl2, respectively (Figure 1B). The commonly used hosts had similar TEs with differences being <4 folds (Figure 1B).

### *E. coli recA*^+^ Strains Have Much Higher TEs and Viable Cell Counts on Agar Plates Than the Corresponding *recA*^−^ Strains

**Figure 2:**
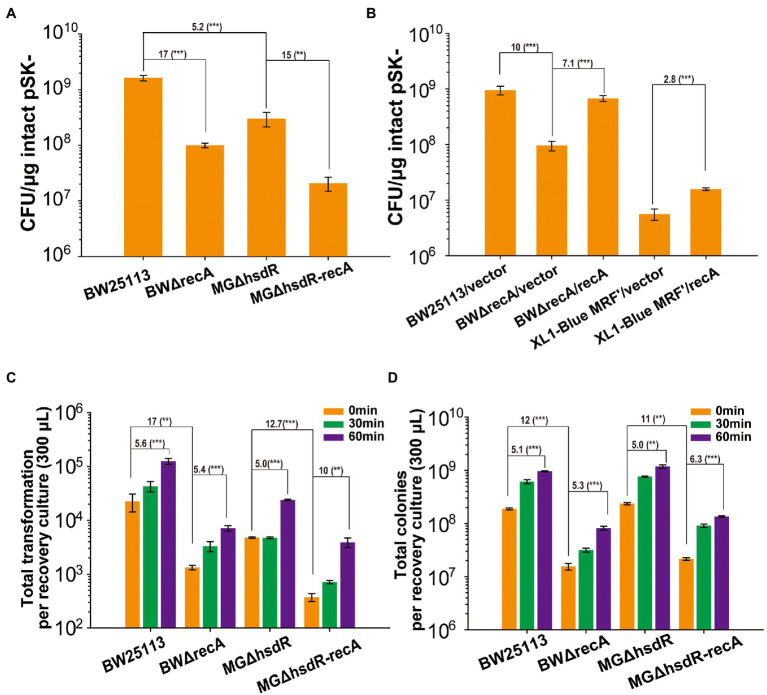
The deletion of *recA* affects TE and cell viability. **(A)** The TEs of competent cells prepared with TSS for three strains and their *recA* mutants. **(B)** The TEs was recovered by complementing the RecA function in the *recA* mutant of BW25113 and XL1-Blue MRF’. **(C)** The transformant colonies of *recA* mutants and their parent wild-type strains formed on LB plates with the Amp antibiotic after recovering in LB medium for 0 to 60 min. **(D)** Total colonies of *recA* mutants and their parent wild-type strains formed on LB plates without antibiotic after recovering in LB medium for 0 to 60 min. Intact pSK- was used for transformation. Unpaired t-tests were performed (** = *p* < 0.01 and *** = *p* < 0.001). The numbers before (***) represent the difference by folds. Data are averages of three samples with standard deviations (error bars).

Since *E. coli* BW25113 is a *recA*^+^ strain, the effect of RecA was tested. The *recA* deletion mutant of *E. coli* BW25113, BWΔrecA, had a TE of 17-fold less than that of BW25113 (Figure 2A). For comparison, *hsdR* was firstly deleted in MG1655 (MGΔhsdR), as BW25113 is *hsdR*^−^. Further deletion of *recA* in MG1655 ΔhsdR (MGΔhsdR-recA) reduced TE by 15 folds (Figure 2A). The *recA* deletion mutants grew slightly slower than their parental strains (Supplementary Figures S1A–C). The reductions in TE were recovered by complementing RecA in BWΔrecA (Figure 2B). When *E. coli* XL1-Blue MRF’ was complemented with *recA* (XL1-Blue MRF’/recA), its TE increased by 3 folds (Figure 2B), but the growth was not increased (Supplementary Figure S1D).

After transformation, the transformants and total live cells in the recovery cultures were determined *via* counting colonies on agar plates with or without the selective antibiotic. At 0 min of recovery, the transformant colonies and total colonies were 17-fold and 12-fold lower for BWΔrecA than those of BW25113, and 13-fold and 11-fold lower for MG1655ΔhsdR-ΔrecA than those of MG1655ΔhsdR (Figures 2C,D). After 60 min of recovery, the number of colonies increased but the trend was the same (Figures 2C,D). Although all cells were collected at the same OD_600_ for competent cells preparation, the wild type with recA had significantly more recovered colonies than their recA mutants on agar plates.

**Figure 3:**
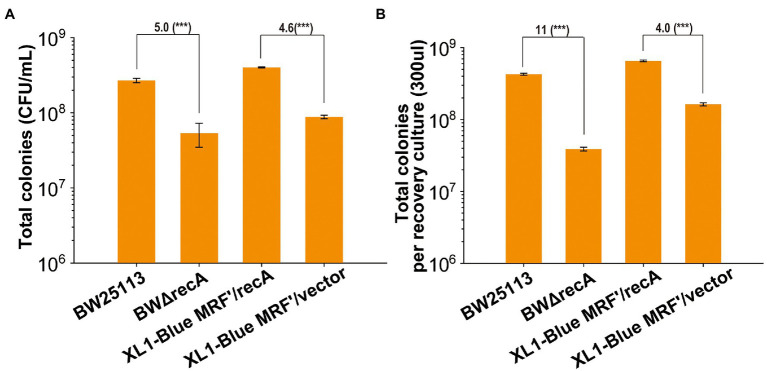
The changes of recovered total colonies for four strains before competent cells preparation and after transformation. **(A)** Total colonies of *recA* mutants and their parent wild-type strains formed on LB plates without antibiotics after they were cultured until their OD_600nm_ reached at 0.5. **(B)** Total colonies of *recA* mutants and their parent wild-type strains formed on LB plates without antibiotics after recovering in LB medium for 60 min. Unpaired t-tests were performed (*** = *p* < 0.001). The numbers before (***) represent the difference by folds. Data are averages of three samples with standard deviations (error bars).

We further tested the number of live cells that formed colonies on LB plates when *E. coli* BW25113, BWΔrecA, XL1-Blue MRF’/vector, and XL1-Blue MRF’/recA grew in LB to OD_600_ of 0.5. BWΔrecA had 5-fold less live cell counts than BW25113, and XL1-Blue MRF’/vector had 4.6-fold less live cell counts than XL1-Blue MRF’/recA (Figure 3A). Further, the total live cell counts in the recovery culture after transformation had an 11-fold difference between BW25113 and BWΔrecA and only a 4-fold difference between XL1-Blue MRF’/vector and XL1-Blue MRF’/recA (Figure 3B). The results indicate that the number of BWΔrecA cells that can form colonies is further reduced after competent cells preparation, storage, and transformation; however, the number of XL1-Blue MRF’ cells is not further reduced. Our results show that the reduced live cells in the *recA*^−^ strains competent cells contribute to the reduced TE. Further, the change of G160 into Asp160 of RecA (the *recA1* mutant) in XL1-Blue MRF’ reduced its viability and TE, but the effect was less severe than the *recA* deletion.

### The Effects of Other Gene Deletions on TE and Growth of *E. coli* BW25113

**Figure 4:**
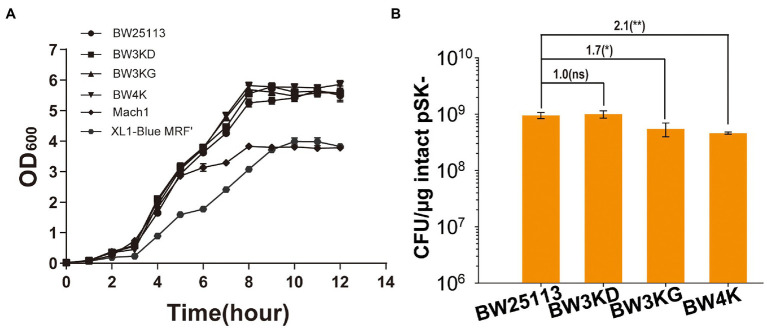
The effect of gene deletion of the growth and TE of *E. coli* BW25113. **(A)** The growth curves of BW25113, BW3KD, BW3KG, and BW4K. The latter two are commercially available cloning strains. **(B)** TEs of these strains. Unpaired t-tests were performed (*** = *p* < 0.001). The numbers before (***) represent the difference by folds. The “ns” shown in the figure means the *p* value is higher than 0.05. The numbers before (**) represent the difference by folds. Data are averages of three samples with standard deviations (error bars).

The common cloning *E. coli* hosts also contain other inactivated genes, including *endA*, *fhuA*, *deoR*, and *galE*, to benefit cloning (van Die et al., 1984; Hanahan et al., 1991; Lin, 1992; Bonhivers et al., 1998; Altermark et al., 2007). Mutants with multiple deletions in *E. coli* BW25113, including BW3KD (ΔendA, ΔfhuA, and ΔdeoR), BW3KG (ΔendA, ΔfhuA, and ΔgalE) and BW4K (ΔendA, ΔfhuA, ΔgalE, and ΔdeoR), were generated. These gene deletions did not affect cell growth (Figure 4A). BW3KD had a similar TE to that of BW25113, and other two mutants with *galE* deletion had TE reduction by about 2 folds (Figure 4B). Thus, the inactivation of these genes is not the reason for the significantly increased TE in BW25113 over the commonly used *E. coli* hosts (Figure 1B).

### *E. coli* BW25113 Has a Significantly Higher Cloning Efficiency Than BWΔrecA and XL1-Blue MRF’

**Figure 5:**
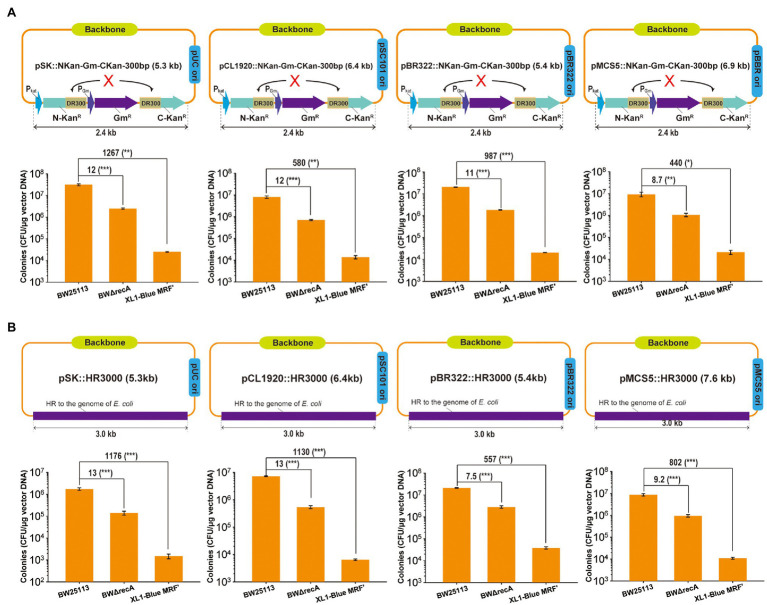
The cloning efficiency by using *E. coli* BW25113, BWΔrecA, and XL1-Blue MRF’ as competent cells. **(A)** Artificial DNA fragment containing 300-bp direct repeats was assembled into plasmids with different replicons. **(B)** A 3 kb-DNA fragment homologous to *E. coli* chromosome was cloned into plasmids with different replicons. The assembled DNA constructs by using TEDA were transformed into the competent cells of three *E. coli* strains prepared with TSS and plated with antibiotic selection. The total colonies (transformants) were counted. The correct constructs were tested by PCR and the positive rates were all higher than 90% (Supplementary Table S3). Data are averages of three samples with standard deviations (error bars). Unpaired t-tests were performed (* = *p* < 0.05; ** = p < 0.01; and *** = *p* < 0.001). Data are averages of three samples with standard deviations (error bars). The positive rates were reported in Supplementary Table S3.

A 2.4-kb fragment carrying the gentamicin resistance gene (Gm^r^) in the middle of the kanamycin resistant gene (Kan^r^) with a 300-bp direct repeat and a 3-kb fragment from BW25113 chromosome were separately ensembled into four plasmids: pBluescript SK- (pSK-), pCL1920, pBR332, and pBBR1MCS-5 (pMCS5; Figures 5A,B). The assembled constructs were transformed into *E. coli* BW25113, BWΔrecA, and XL1-Blue MRF’. The cloning efficiency was defined as the total number of colonies (transformants) recovered on selective agar plates. The cloning efficiency with BW25113 was about 7- to 13-fold higher than those with BWΔrecA and 440- to 1,267-fold higher than those with XL1-Blue MRF’ (Figures 5A,B). When 20 or more colonies were checked by PCR, positive cloning rates were all higher than 90% (Supplementary Table S3). Thus, the cloning efficiencies are positively correlated with the TEs.

### The Effect of RecA on Insert Stability Is Not Observed

**Figure 6:**
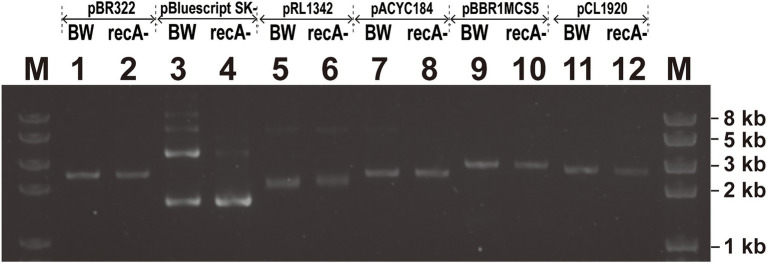
The screen of plasmids multimerization with different replicons in *E. coli* BW25113 and *E. coli* BWΔrecA. The top two lines on the gel showed the plasmids that were transformed into either BW25113 (BW) or BWΔrecA (*recA*^−^). Note: The two lower bands in Lanes 3 and 4 are the relaxed form and supercoiled form of pBluescipt SK- monomer; the faint bands on top of Lane 3 are the dimer forms.

Intermolecular recombination between copies of the plasmid renders dimerization (Boe and Tolker-Nielsen, 1997). When pSK- was extracted from BW25113 and BWΔrecA and analyzed through gel electrophoresis, the two bright bands were the supercoiled and relaxed forms of pSK- isolated from BW25113 and BWΔrecA (Figure 6, lanes 3 and 4), and the faint bands on top were the dimer form of pSK^−^ from BW25113 (Figure 6, lane 3). The results indicate that a small fraction of the high copy number plasmid pSK- is in the dimer form in BW25113 but not in BWΔrecA. Dimerization was not observed with the medium copy number plasmids pBR322 and pMCS5 and the low copy number plasmid pCL1920 in both BW25113 and BWΔrecA (Figure 6).

**Figure 7:**
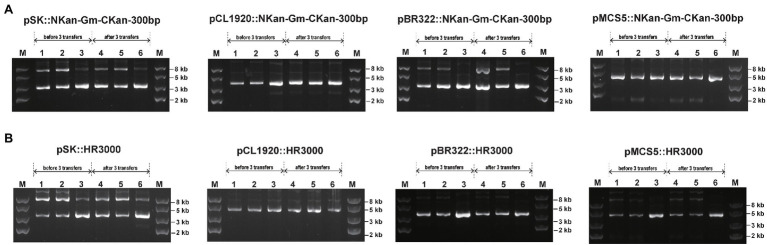
The stability of plasmids with homology features after extended culturing for 3 transfers. Three transformants were randomly selected from plates and cultured in LB medium for 3 transfers, representing about 60 generations. The plasmids were then extracted and checked *via* gel electrophoresis. **(A)** NKan-Gm-CKan-300 bp was assembled into four vectors. **(B)** HR300 was assembled into four vectors. Lanes 1–3 in each figure represent plasmids extracted from three cultures before 3 transfers, and lanes 4–6 in each figure represent plasmids extracted from three cultures after 3 transfers. Lane 1–2 and 4–5 represent plasmids extracted from strain BW25113, and Lane 3 and 6 represent plasmids extracted from strain XL1-Blue MRF’.

To test whether recombination occurred between direct repeats on plasmids, the four plasmids carrying Gm^r^ inserted in the middle of Kan^r^ with the 300-bp direct repeats in BW25113 and BWΔrecA were analyzed (Figure 5A). When recombination occurred between the direct repeats, the antibiotic resistance would be altered from Kan^−^/Gm^+^ into Kan^+^/Gm^−^ (Figure 5A). Three transformant colonies were randomly selected and cultured in LB without antibiotics. After 3 transfers (>60 generations), no Kan^+^ colonies were obtained, and the plasmids were the same *via* gel electrophoresis analysis (Figure 7A); the plasmid yields from 3 ml of overnight cultures were also similar (Supplementary Table S4).

To check whether recombination occurred between the identical fragments on plasmids and the chromosome, a 3,000-bp DNA sequence from the genome of BW25113 was amplified and assembled into the four plasmids (Figure 5B). Three transformant colonies were randomly selected and cultured in LB with the proper antibiotics. After 3 transfers, the plasmids were extracted and analyzed by electrophoresis, no plasmid loss was observed (Figure 7B; Supplementary Table S4).

**Figure 8:**
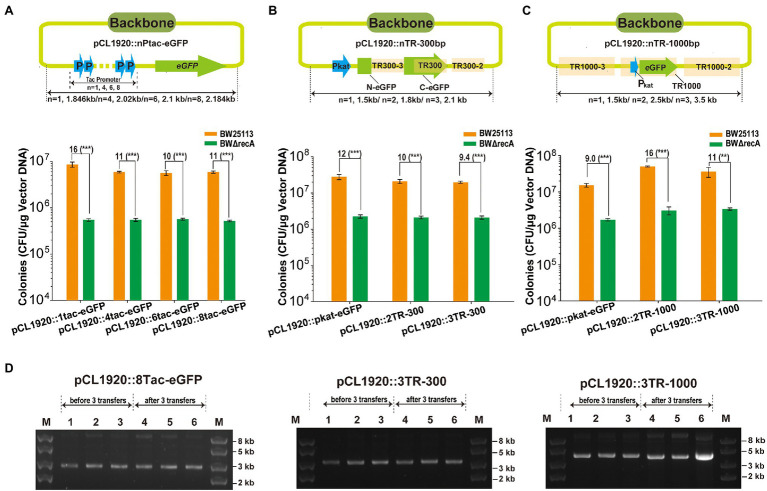
The cloning of plasmids with TRs at different lengths in *E. coli* BW25113. **(A)** DNA fragments containing an eGFP gene under the control of 1, 4, 6, and 8 units of the 41-bp Ptac was assembled into pCL1920. **(B)** DNA fragments containing 1, 2, or 3 units of the 300-bp TRs (TR300), derived from the C-terminal eGFP gene, were assembled into pCL1920. **(C)** DNA fragments containing 1, 2, or 3 units of the 1,000-bp TRs (TR1000), included the Pkat and eGFP, were assembled into pCL1920. The constructs were transformed into *E. coli* BW25113 and BW25113ΔrecA, and the total transformant colonies on LB plates with antibiotic selection were counted. Unpaired t-tests were performed (*** = p < 0.001). The numbers before (***) represent the difference by folds. Data are averages of three samples with standard deviations (error bars). **(D)** Gel electrophoresis of pCL1920::8Tac-eGFP, 3TR-300 bp, and 3TR-of 1,000 bp. Lanes 1–3 in each figure represent plasmids extracted from the three cultures before 3 transfers, and lanes 4–6 in each figure represent plasmids extracted from the three cultures after 3 transfers. Lane 1–2 and 4–5 represent plasmids extracted from BW25113, and Lane 3 and 6 represent plasmids extracted from the strain BWΔrecA.

To check whether homologous recombination occurred among tandem repeats on plasmids, the repeats with unit length at 41-, 300-, and 1,000-bp were assayed. First, the 4, 6, and 8 tandem repeats of the 41-bp tac promoter, Ptac (TR41), with the eGFP gene were amplified and assembled into pCL1920, and the assembly of a single Ptac before eGFP was assembled into pCL1920 (pCL1920::1tac-eGFP) as the control (Figure 8A). The 300-bp tandem repeats and 1,000 bp tandem repeats were generated by using the plasmid pCL1920::Pkat-eGFP as the backbone (Figures 8B,C). The 2 and 3 tandem repeats at length of 300 bp and 1,000 bp were reassembled with pCL1920, and the assembly of single unit of the repeats was used as the control (Figures 8B,C). The presence of tandem repeats with all three lengths did not change the assembly efficiency for both BW25113 and BWΔrecA (Figures 8A–C). The positive rates for the assembly of all tandem repeats were higher than 90% for both strains, except the assembly of three units of TR1000. The positive rates were 80% in BWΔrecA and 50% in BW25113; however, 11-fold more transformant colonies were obtained with BW25113. All these constructed plasmids with highly tandem repeats including the one with 3 units of TR1000 repeats could be stably maintained in cells of BW25113 without apparent recombination or plasmid loss after three transfers in LB with antibiotic selection (Figure 8D; Supplementary Table S4). Collectively, these results suggest that these plasmids with homologous features were successfully maintained in the *recA*^+^ BW25113.

## Discussion

Our results support the use of the TSS method to prepare *E. coli* BW25113 and its *recA* deletion mutant BWΔrecA cells for cloning and DNA assembly (Xia et al., 2015, 2019). The choice of host strains is heavily relying on their availability. During our investigation on the complete pathway of the recombination of short-homologous ends in *E. coli* (Yang et al., 2021), we noticed *E. coli* BW25113 may be efficient in cloning and DNA assembly. The significantly higher TE and cloning efficiency of *E. coli* BW25113 over commonly used *E. coli* strains are beyond our expectations (Figures 1, 5). Even its *recA* deletion mutant BWΔrecA is significantly better, with its TE and cloning efficiency being 12- and >44-fold higher than those of *E. coli* XL1-Blue MRF’ (Figures 2, 5). The difference is enhanced by using the TSS method over the Hanahan and Inoue methods (Figure 1). However, the Hanahan and Inoue methods are usually better in preparing competent cells than the TSS methods for commonly used *E. coli* hosts, such as *E. coli* XL1-Blue MRF’ (Figure 1; Hanahan et al., 1991; Xia et al., 2019). Competent cells prepared with the Hanahan and Inoue methods may reach the TEs of 10^9^ CFU/μg DNA with supercoiled plasmids (Inoue et al., 1990; Hengen, 1996; Xia et al., 2019), but both methods are normally kept the TEs at 10^8^ CFU/μg DNA (Green and Sambrook, 2018, 2020). The TSS method is also commonly used because of its simplicity, in which the harvested cells are resuspended in TSS, stored at −80°C, and used for transformation, but its TS is around 10^7^ CFU/μg DNA (Chung et al., 1989; Parrish et al., 2004; Xia et al., 2019). In our hands, the commonly used *E. coli* cloning strain XL1-Blue MRF’ had TEs around 10^7^ CFU/μg DNA by using the three methods (Figure 1A), and *E. coli* BW25113 had TE higher than 10^9^ CFU/μg DNA (Figure 1A). Although electroporation can reach up to 10^10^ CFU/μg DNA with supercoiled plasmids (Dower et al., 1988), it is not frequently used with the *in vitro* methods. The limitation is related to arching when the assembled DNA is directly used in large volumes (Sambrook et al., 2017). To prevent arching, either the assembled DNA is purified or a small volume is directly used in electroporation. The DNA loss during purification or small volumes may lead to low cloning efficiency (Tan and Yiap, 2009). Since both *E. coli* BW25113 and BWΔrecA are widely available (Baba et al., 2006), their chemically prepared competent cells by using the TSS method may be the better choices to be used with the *in vitro* methods for efficient cloning or DNA assembly.

*E. coli* BW25113 displays at least 100-fold higher TE than commonly used *E. coli* cloning strains (Figure 1B). RecA is a major factor contributing to the high TE and cloning efficiency in *E. coli* BW25113 (Figures 1–3, 5, 8), as its deletion reduced TE of BWΔrecA by 16 folds (Figure 2A), which is still 6-, 12-, 10-, 16-, 17-, and 23-fold higher than that of Omnimax, XL1-Blue MRF’, Stbl3, DH5α, Mach1, and Stbl2, respectively (Figures 1B, 2A). Therefore, RecA accounts for about half of the increased TE in *E. coli* BW25113. The *recA*^−^ has been reported to reduce the mutant’s growth (Cox et al., 2008), but the decreases for both BW25113 and MG1655 were minor (Supplementary Figures S1A,B). The reduced TE in *recA*^−^ strains is likely due to the reduced abilities of the *recA*^−^ strains to form colonies on LB plates. The *recA*^−^ strains had about 5-fold fewer colonies on LB plates than the *recA*^+^ strains when they grew to the same OD_600nm_ (Figure 3A). The colonies of the *recA*^−^ strains were further reduced to 17-fold and 13-fold fewer than that of the corresponding *recA*^+^ strains (BW25113 and MG1655) after competent cell preparation, storage, and transformation (Figure 2C). *E. coli* XL1-Blue MRF’ contains *recA*1 that has a point mutation changing Gly160 to Asp160 (Bullock, 1987). *E. coli recA1* mutants are defective in recombination, as the RecA1 protein binds to single-stranded DNA but cannot carry out the ATP-dependent strand exchange reaction (Clark and Margulies, 1965; Muench and Bryant, 1990). *E. coli* XL1-Blue MRF’/*recA* only increased TE and the total colony counts by about 3–4 folds over XL1-Blue MRF’ (Figures 2B, 3B). The reduced increase is likely due to the interference of RecA1 in *E. coli* XL1-Blue MRF’/*recA*. Our results show a positive correlation between the TEs and the total live cells that are able to form colonies on LB plates after transformation. The reduced colony numbers of the *recA*^−^ strains could be due to the presence of H_2_O_2_ in agar plates from autoclaving (Tanaka et al., 2014), which damages DNA (Hong et al., 2019). RecA is the core component of bacterial DNA homologous recombination (Kowalczykowski, 2000; Morimatsu and Kowalczykowski, 2014), participating in DNA damage repair (Li and Heyer, 2008). Hence, RecA enhances cell survival in the presence of H_2_O_2_. The mechanism warrants further investigation.

Other intentionally inactivated genes, such as *galE*, may contribute to the reduced TE but have little effect (Figure 4B). *E. coli* BW25113 may represent a good host for cloning for its intrinsic high TE when prepared with the TSS method (Figures 1, 2). *E. coli* BW25113 had 5-fold higher TE than MGΔhsdR, which is the *hsdR* deletion strain of *E. coli* MG1655 (Figure 2A). Only few differences exist on their genomes (Grenier et al., 2014). MGΔhsdR contains mutations in some phosphotransferase systems (Grenier et al., 2014), and BW25113 contains a mutation in the YjjP membrane protein (Grenier et al., 2014; Price et al., 2016), which is related to the transport of succinate (Fukui et al., 2017). There are also some differences in mobile elements between MGΔhsdR and BW25113, which may affect the stability of DNA (Darmon and Leach, 2014). These mutations do not affect the growth of the two strains in LB (Supplementary Figures S1A,B). It is unclear which difference determines their TEs. Further study is needed to reveal the mutations that affect the TEs of these strains.

Although it is believed that the RecA-dependent recombination affects plasmid stability, clear evidence is only related to the dimer formation with high copy number plasmids, which leads to plasmid loss without selection (Herman-Antosiewicz and Wegrzyn, 1999; Summers et al., 2010; Standley et al., 2019). We also observed dimers with plasmids harbor pUC18 ori, but the plasmids were stably maintained with selection (Figures 6, 7; Supplementary Table S4). When the monomer and dimer mixture isolated from BW25113 was transformed into BWΔrecA or XL1-Blue MRF’, only monomers were recovered, possibly due to RecF that is known to resolve dimers back to monomers (Summers and Sherratt, 1984). Further, *recA*^+^ significantly increased the cloning efficiency (Figures 5, 8) but did not affect the stability of inserts with tandem repeats of various size and a 3-kb host DNA (Figures 5, 7, 8). Considering the rate of RecA-dependent homologous recombination in *E. coli* is very low, occurring at approximately 10^−5^ per cell generation (Lovett et al., 2002), the effect of RecA on cloned inserts may be tolerated. Indeed, the *recA*^+^ *E. coli* BL21(DE3) is routinely used in protein overexpression (Chan et al., 2013), and *recA*^+^ K-12 derivatives are widely used in metabolic engineering (Cui et al., 2018; Badri et al., 2021). Hence, the effect of RecA on insert stability will not affect the cloned inserts in most cases, especially when homologous regions are small or not present (Lovett et al., 2002).

In summary, *E. coli* BW25113, a *recA*^+^ strain, is >100-fold more efficient for transformation than the commonly used *E. coli recA*^−^ cloning strains when the competent cells are prepared with the TSS method (Figure 1B). The difference in cloning efficiency between BW25113 and XL1-Blue MRF’ is more dramatic, higher than 440 folds (Figure 5). RecA plays a major role in the increased TE and cloning efficiency (Figures 2, 5). The deletion of *recA* significantly reduces the fitness of the *recA*^−^ strains, as they form less colonies than their corresponding *recA*^+^ strains on LB plates at the same OD_600nm_. Since we did not observe apparent insert instability with several homologous features in different vectors (Figures 5, 7, 8), *E. coli* BW25113 and other *recA*^+^ strains may be reconsidered for use in cloning for significantly increased success rates, especially when the inserts have none or minimal homologous features. When recA becomes a real concern, the *recA* mutant *E. coli* BWΔrecA can be used, as it is still significantly more efficient in cloning and DNA assembly than the commonly used cloning strains. BW25113 and its single-gene deletion mutants are readily available (Baba et al., 2006). They may be used to increase the cloning success rates in DNA assembly.

## Data Availability Statement

The original contributions presented in the study are included in the article/**Supplementary Material**, further inquiries can be directed to the corresponding authors.

## Author Contributions

YY acquired and analyzed the data. QY contributed to the preparation of competent cells. YY and MW contributed to the gene deletion and plasmid construction. YY and RZ contributed to the test of plasmid instability in RecA^+^ cells. YX, LX, and HL conceptualized this project and supervised the overall experiments. YY, YX, and HL prepared the manuscript. All authors contributed to the article and approved the submitted version.

## Funding

We appreciated supports by grants from the Natural Science Foundation of China (31870085, 91951202, and 31961133015), the funding by the State Key Laboratory of Microbial Technology, and Qilu Youth Scholar Startup Funding of SDU (to YX).

## Conflict of Interest

The authors declare that the research was conducted in the absence of any commercial or financial relationships that could be construed as a potential conflict of interest.

## Publisher’s Note

All claims expressed in this article are solely those of the authors and do not necessarily represent those of their affiliated organizations, or those of the publisher, the editors and the reviewers. Any product that may be evaluated in this article, or claim that may be made by its manufacturer, is not guaranteed or endorsed by the publisher.

## Supplementary Material

The Supplementary Material for this article can be found online at: https://www.frontiersin.org/articles/10.3389/fmicb.2022.838698/full#supplementary-material
