# Supplementary material for: Escherichia coli BW25113 Competent Cells Prepared Using a Simple Chemical Method Have Unmatched Transformation and Cloning Efficiencies
Source: Front Microbiol. 2022 Mar 24;13:838698. doi: 10.3389/fmicb.2022.838698 (PMC8989280; doi:10.3389/fmicb.2022.838698)
Supplement: Supplementary file 1 [file Data_Sheet_1.pdf]

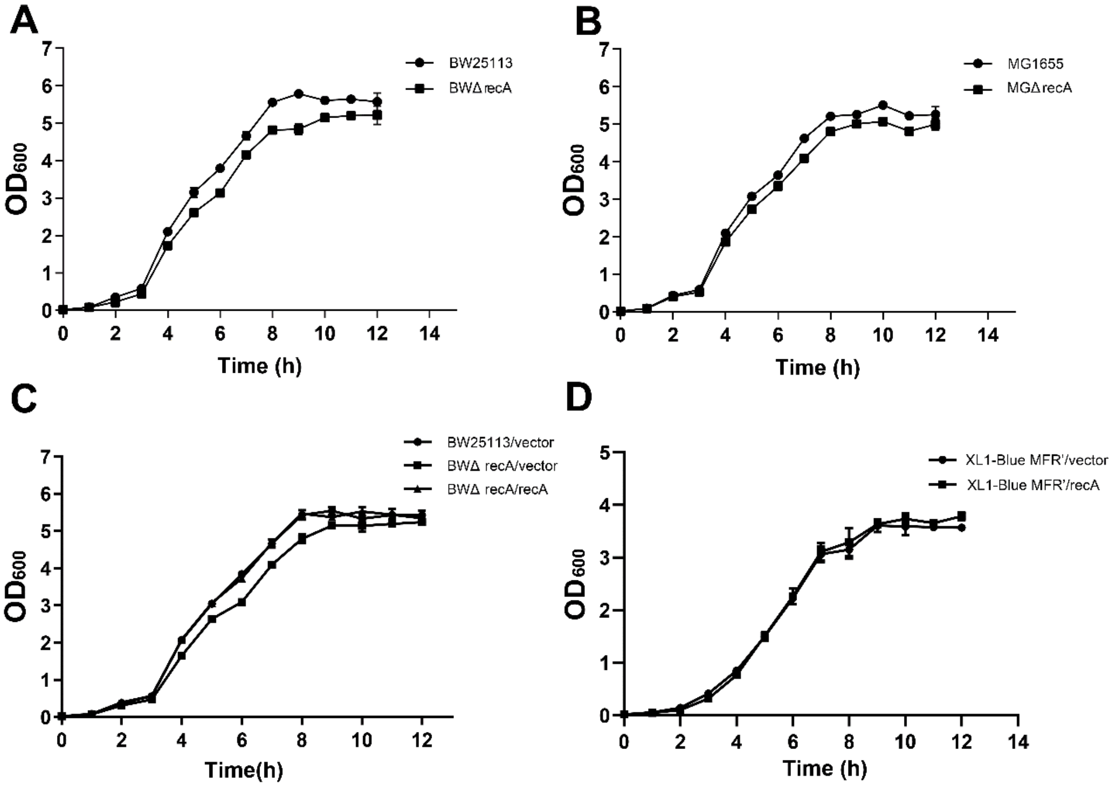

Supplementary Figure S1. The growth curves of different strains. A) The growth curves of E. coli BW25113, BWΔrecA. **B)** The growth curves of *E. coli* MGΔhsdR and MGΔhsdR-recA. **C)** The effects of RecA complementation to the growth of BWΔrecA. **D)** The effects of RecA complementation to the growth of XL1-Blue MRF'. Data are averages of three samples with standard deviations (error bars).

Supplementary Table S1. The strains and plasmids used in this study

Strains and Plasmids	Functions and features	Origin
***E. coli* strains**		
XL1-Blue MRF’	Δ(*mcrA*)183 Δ(*mcrCB-hsdSMR-mrr*)173 *endA1 supE44 thi-1 recA1 gyrA96 relA1* lac *[F’ proAB* $lacI^{q}Z\Delta M15$ *Tn10* $(Tet^{r})]$	Agilent
MG1655	K-12 $F^{–}$ $\lambda^{–}$ $ilvG^{–}$ *rfb-50 rph-1*	NBRP E.coli, Japan
W3110	F^{-} λ^{-} *rph-1* INV(*rrnD, rrnE*)	Coli Genetic Stock Center (CGSC)
BW25113	$LacI^{+}$ $rrnB_{T14}$ $\Delta lacZ_{WJ16}$ *hsdR514* $\Delta araBAD_{AH33}$ $\Delta rhaBAD_{LD78}$ *rph-1 Δ(araB–D)567 Δ(rhaD–B)568 ΔlacZ4787(::rrnB-3) hsdR514 rph-1*	CGSC
Mach1	Str.W Δ*recA1398 endA1 fhuA* Φ80Δ(*lac*)M15 Δ(*lac*)*X*74 hsdR($r_{K}^{–}$ m_{K}^{+})	Invitrogen
Omnimax2	F′[$proAB^{+}$ $lacI^{q}$ *lacZΔM15 Tn10*(Tet^{R}) Δ(*ccdAB*)] *mcrA* Δ(*mrr-hsdRMS-mcrBC) φ80(lacZ)ΔM15 Δ(lacZYA-argF)U169 endA1 recA1 glnV44 thi-1 gyrA96*(Nal^{R}) *relA1 tonA panD*	Invitrogen
Stbl2	*F- endA1 glnV44 thi-1 recA1 gyrA96 relA1 Δ(lac-proAB) mcrA Δ(mcrBC-hsdRMS-mrr)* λ^{-}	Invitrogen
Stbl3	F^{-} *mcrB mrr hsdS20* (r_{B}^{-}, m_{B}^{-}) *recA13 supE44 ara-14 galK2 lacY1 proA2 rpsL20* (Str^{R}) *xyl-5* λ^{-} *leu mtl-1*	Invitrogen
**Mutant of MG1655**		
ΔhsdR	The *recA* gene deletion mutant of *E. coli* MG1655	This study
ΔhsdR-recA	The genes *hsdR* and *recA* were deleted in *E. coli* MG1655	
**Mutant of W3110**		
ΔhsdR	The *recA* gene deletion mutant of *E. coli* W3110	This study
ΔhsdR-recA	The genes *hsdR* and *recA* were deleted in *E. coli* W3110	This study
**Mutants of BW25113**		

BWΔrecA	The *recA* gene deletion mutant of *E. coli* BW25113	
BW3KG	ΔendA, ΔfhuA, ΔgalE	This study
BW3KD	ΔendA, ΔfhuA, ΔdeoR	This study
BW4K	ΔendA, ΔfhuA, ΔgalE, ΔdeoR	This study
**Strains with Plasmids**		
BW25113/vector	BW25113 harboring pCL1920	This study
BWΔrecA/vector	BWΔrecA harboring pCL1920	This study
BWΔrecA/recA	BWΔrecA harboring pCL1920-Pnat-recA	This study
XL1-Blue MRF'/vecor	XL1-Blue MRF' harboring pCL1920	This study
XL1-Blue MRF'/recA	XL1-Blue MRF' harboring pCL1920-Pnat-recA	This study
**Plasmids**		
pBluescript SK$^-$	Abbreved as pSK-, pUC18 ori, Amp^R, used to test TE of competent cells	Stratagene
pCL1920	pSC101 ori, Spc^R	Addgene
pBBR1MCS5	Abbreved as pMCS5, pBBR ori, Gm^R	Addgene
pBR322	pBR322 ori, Amp^R and Tet^R	Addgene
pACYC184	p15A ori, Cmr^R and Tet^R	Addgene
pKD4	R6K ori, Kan^R and Amp^R, used for gene deletion in *E. coli*	Addgene
pTKred	pSC101 ori, temperature sensitive, Spc^R, used for gene deletion in *E. coli*	Addgene
pCP20	pSC101 ori, temperature sensitive, Amp^R, used for gene deletion in *E. coli*	Addgene
pSK::Pkat-eGFP	derived from pSK-, phbCAB genes under the control of 5 tac promoters	This study
pCL1920::Pnat-recA	The *recA* gene of *E. coli* was cloned in pCL1920 with its native promoter (Pnat)	This study
pCL1920::NKan-Gm-CKan-300	Plasmid pCL1920 was introduced with 300 bp direct repeat, used to test intramolecular recombination occurring on plasmid DNA	This study
pBR322::NKan-Gm-CKan-300	Plasmid pBR322 was introduced with 300 bp direct repeat, used to test intramolecular recombination occurring on plasmid DNA	This study
pBBR1MCS5::NKan-Gm-CKan-300	Plasmid pMCS5 was introduced with 300 bp direct repeat, used for test intramolecular recombination occurring on plasmid DNA	This study
pSK::NKan-Gm-CKan-300	Plasmid pSK- was introduced with 300 bp direct repeat, used for test intramolecular recombination occurring on plasmid DNA	This study

pCL1920::HR3000	Plasmid pCL1920 cloned with 3000 bp sequence with homology to BW25113 genome, used to test the recombination occurring between plasmid and its chromosome	This study
pBR322::HR3000	Plasmid pBR322 cloned with 3000 bp sequence with homology to BW25113 genome, used to test the recombination occurring between plasmid and its chromosome	This study
pMCS5::HR3000	Plasmid pMCS5 cloned with 3000 bp sequence with homology to BW25113 genome, used to test the recombination occurring between plasmid and its chromosome	This study
pSK::HR3000	Plasmid pMCS5 cloned with 3000 bp sequence with homology to BW25113 genome, used to test the recombination occurring between plasmid and its chromosome	This study
pCL1920::2TR-300	Plasmid pCL1920::Pkat-eGFP was introduced two 300 bp tandem repeats, used for test intramolecular recombination occurring on plasmid DNA	This study
pCL1920::3TR-300	Plasmid pCL1920::Pkat-eGFP was introduced three 300 bp tandem repeats, used for test intramolecular recombination occurring on plasmid DNA	This study
pCL1920::2TR-1000	Plasmid pCL1920::Pkat-eGFP was introduced two 300 bp tandem repeats, used for test intramolecular recombination occurring on plasmid DNA	This study
pCL1920::3TR-1000	Plasmid pCL1920::Pkat-eGFP was introduced three 300 bp tandem repeats, used for test intramolecular recombination occurring on plasmid DNA	This study

Supplementary Table S2. The oligoes used in this study

NO.	Primers	SEQUENCES	Purposes and characteristics
1.	recA-del-fr	GAACATATTGACTATCCGGTATTACCCGGCATGACAGGAGTAAAAATGGCTCAGCATTACACGTCTTGAGCGAT	Primers used to amplify DNA fragments from pKD4, and used to knock out the *recA* gene
2.	recA-del-rev	CAGATGCGACCCTTGTGTATCAAACAAGACGATTAAAAATCTTCGTTAGTGGAACACTTAACGGCTGACATG	
3.	recA-out-fr	TACCGATATTGCCGGTAGCT	Primers used to check if *recA* gene was successfully deleted
4.	recA-out-rev	GTCGATGTCACATTCACGCAT	
5.	endA-del-fr	CAGCTTTCGCTACGTTGCTGGCTCGTTTTAACACGGAGTAAGTGATGTACCAGCATTACACGTCTTGAGCGAT	Primers used to amplify DNA fragments from pKD4, and used to knock out the *endA* gene
6.	endA-del-rev	GGGGTTAACAAAAAGAATCCCGCTAGTGTAGGTTAGCTCTTTCGCGCCTGGGAACACTTAACGGCTGACATG	
7.	endA-fr-out	GTGGTACCGCACGAACTGGCA	Primers used to check if *endA* gene was successfully deleted
8.	endA-rev-out	GCGACATCACCTGACCGAGG	
9.	fhuA-del-fr	ATTCTCGTTTACGTTATCATTCACTTTACATCAGAGATATACCAATGGCGCAGCATTACACGTCTTGAGCGAT	Primers used to amplify DNA fragments from pKD4, and used to knock out the *fhuA* gene
10.	fhuA-del-rev	TGGGCACGGAAATCCGTGCCCCAAAAGA	

		GAAATTAGAAACGGAAGGTTGCGGAACACTTAACGGCTGACATG	
11.	fhuA-out-fr	TGTGCCAGCAGAGCGAGATG	Primers used to check if *fhuA* gene was successfully deleted
12.	fhuA-out-rev	ACCAGTTCACGCACGGTCA	
13.	galE-del-fr	TTATGCTATGGTTATTTCATACCATAAGCCTAATGGAGCGAATTATGAGACAGCATTACACGTCTTGAGCGAT	Primers used to amplify DNA fragments from pKD4, and used to knock out the *galE* gene
14.	galE-del-rev	TCAACGGGATTAAATTGCGTCATGGTCGTTCCTTAATCGGGATATCCCTGGGAACACTTAACGGCTGACATG	
15.	galE-out-fr	GATTAGCGAAGGTGAAACG	Primers used to check if *galE* gene was successfully deleted
16.	galE-out-rev	GATTTCCGTCAATGCTGCA	
17.	deoR del-Fr	AGTGTAGTATTGAGCGGCTCGCTTCAATAACTATTCAGAGGGATTATGGAACAGCATTACACGTCTTGAGCGAT	Primers used to amplify DNA fragments from pKD4, and used to knock out the *deoR* gene
18.	deoR del-rev	GATGGCGCGAAACGTCATCCGGTTATACGTCATTAATACATCAACTTAATGGAACACTTAACGGCTGACATG	
19.	deoR -out-fr	GCGATCACGGTACGGTGAT	Primers used to check if *deoR* gene was successfully deleted
20.	deoR -out-rev	CTCAGTGACCATACCGCGT	
21.	pcl1920-fr-Pnp	GATCCTCTAGAGTCGACCTGCAG	Primers used to amplify linearized vector with

22.	pcl1920-rev-Pnp	GATCGCGTATGCCGCCATG	pCL1920 as template, which was used to construct pCL1920::Pnp-recA
23.	pnp-fr-recA	CATGGCGGCATACGCGATCACGCGGATTTGTCACCTACAG	Primers used amplify F-recA with its native promoter and use BW25113 genome as template, which was used to construct pCL1920::Pnp-recA
24.	pnp-rev-recA	CAGGTCGACTCTAGAGGATCTTAAAAATCTTCGTTAGTTTCTGCTACGCC	
25.	psk-kan-F	GCGGGACTCTGGGGTTCGAAATG	Primers used amplify linearized vector with pSK-Kan (Xia et al., 2019) as template, which was used for pSK::Pkat-eGFP cloning
26.	psk-kan-R	GCGAAACGATCCTCATCCTGTCTCT	
27.	Pkat-eGFP-F30	GGCGGCCGCTCTAGAACTAGTGGATCCCCCACTGGGCTATCTGGACAAGGG	Primers used amplify Pkat-eGFP fragment with SmaI treated pSK::Pkat-eGFP (Xia et al., 2019) as template, which was used for pSK::Pkat-eGFP cloning with 30 bp homologous ends
28.	Pkat-eGFP-R30	GATAAGCTTGATATCGAATTCCTGCAGCCCGCATTCTGCCGACATGGAA	
29.	pMCS5-fr-spc	CGGCAAATAACAATTCGTTCAAGCCGAGATC	Primers used amplify linearized vector with pBBR1MCS5 as template, which was used to construct pBBR1MCS5::Spc
30.	pMCS5-rev-spc	CTTCCCTCATCGTTGCTGCTCCATAACATCA	
31.	spc-fr-pMCS5	AGCAGCAACGATGAGGGAAGCGGTGATCGC	Primers used amplify F-spc fragment with pCL1920 as template, which was used to construct pBBR1MCS5::Spc
32.	spc-rev-pMCS5	GAACGAATTGTTATTTGCCGACTACCTTGGTGATCT	
33.	NKan-fr	GGGAAAACGCAAGCGCAAAGAGAAAG	Primers used amplify F-NKan fragment with pKD4 as template, which was used to construst NKan-Gm-CKan-300bp fragment
34.	NKan-rev	GCTTCCATGTCGGCAGAATGCTACGTGCTCGCTCGATGCGA	
35.	Gm-fr	AGCATTCTGCCGACATGGAAGC	Primers used amplify F-Gm fragment with pBBR1MCS5 as template, which was used to
36.	Gm-rev	GACAGGTCGGTCTTGACAAAAAGAACC	

		GGCGTTGTGACAATTTACCG	construst NKan-Gm-CKan-300bp fragment
37.	CKan-fr	GTTCTTTTTGTCAAGACCGACCTGTCC	Primers used amplify F-CKan fragment with pKD4 as template, which was used to construst NKan-Gm-CKan-300bp fragment
38.	CKan-rev	CATAGAAGGCGGCGGTGGAA	
39.	pcl1920-fr	TTAAGCCAGCCCCGACACC	Primers used amplify linearized vector with pCL1920 as template, which was used to construct pCL1920::NKan-Gm-CKan-300 and pCL1920::HR3000
40.	pcl1920-rev	CTGTCGTGCCAGCTGCAT	
41.	NKan-Gm-CKan-300-fr-pCL1920	TAATGCAGCTGGCACGACAGGGGAAAACGCAAGCGCAAAGAGAAAGC	Primers used amplify NKan-Gm-CKan-300bp fragment with F-NKan, F-Gm and F-CKan as template, which was used to construst pCL1920::NKan-Gm-CKan-300
42.	NKan-Gm-CKan-300-rev-pCL1920	GGGTGTCGGGGCTGGCTTAACATAGAAGGCGGCGGTGGAATC	
43.	HR3000-fr-pcl1920	TAATGCAGCTGGCACGACAGATGCCTTTTACACTTGGTCAACGC	Primers used amplify HR3000 fragment with BW25113 genome as template, which was used to construst pCL1920::HR3000
44.	HR3000-rev-pcl1920	GGGTGTCGGGGCTGGCTTAACTCGGCTTGTTGACCACCAT	
45.	pBR322-fr	GGCACCTCGCTAACGGATTCA	Primers used amplify linearized vector with pBR322 as template, which was used to construct pBR322::NKan-Gm-CKan-300 and pBR322::HR3000
46.	pBR322-rev	GTGATACGCCTATTTTTATAGGTTA	
47.	NKan-Gm-CKan-300-fr-pBR322	TATAAAAATAGGCGTATCACGGGAAAACGCAAGCGCAAAGAGAAAGC	Primers used amplify NKan-Gm-CKan-300bp fragment with F-NKan, F-Gm and F-CKan as template, which was used to construst pBR322::NKan-Gm-CKan-300
48.	NKan-Gm-CKan-300-rev-pBR322	GAATCCGTTAGCGAGGTGCCCATAGAAGGCGGCGGTGGAATC	
49.	HR3000-fr-pBR322	GTGACCGCGCGCTTTC	Primers used amplify HR3000 fragment with BW25113 genome as template, which
50.	HR3000-rev-pBR322	CATTGCTCACCTCTCAACAC	

			was used to construst pBR322::HR3000
51.	pSK-fr	GGGCTGCAGGAATTCGATATCAAG	Primers used amplify linearized vector with pBluescript SK$^-$ as template, which was used to construct pSK::NKan-Gm-CKan-300
52.	pSK-rev	GGGGGATCCACTAGTTCTAGAGC	
53.	NKan-Gm-CKan-300-fr-pSK	CTAGAACTAGTGGATCCCCCGGGAAAACGCAAGCGCAAAGAGAAAGC	Primers used amplify NKan-Gm-CKan-300bp fragment with F-NKan, F-Gm and F-CKan as template, which was used to construst pSK::NKan-Gm-CKan-300
54.	NKan-Gm-CKan-300-rev-pSK	ATATCGAATTCCTGCAGCCCCATAGAAGGCGGCGGTGGAATC	
55.	pSK-fr-HR3000	GGGCTGCAGGAATTCGATATCAAG	Primers used amplify linearized vector with pBluescript SK$^-$ as template, which was used to construct pSK::HR3000
56.	pSK-rev-HR3000	CTGGCCGTCGTTTTACAACG	
57.	HR3000-fr-pSK	CGTTGTAAAACGACGGCCAGATGCCTTTTACACTTGGTCAACGC	Primers used amplify HR3000 fragment with BW25113 genome as template, which was used to construst pSK::HR3000
58.	HR3000-rev-pSK	ATATCGAATTCCTGCAGCCCCTCGGCTTGTTGACCACCAT	
59.	pMCS5::Spc-fr	GCGTTAATATTTTGTTAAAATTCGCG	Primers used amplify linearized vector with pBBR1MCS5::Spc as template, which was used to construct pBBR1MCS5-Spc::NKan-Gm-CKan-300 and pBBR1MCS5-Spc::HR3000
60.	pMCS5::Spc-rev	TAAGCATTCTGCCGACATGGA	
61.	NKan-Gm-CKan-300-fr-pMCS5	CTGGTGCTGGGATTATGATG	Primers used amplify NKan-Gm-CKan-300bp fragment with F-NKan, F-Gm and F-CKan as template, which was used to construst pBBR1MCS5-Spc::NKan-Gm-CKan-300
62.	NKan-Gm-CKan-300-rev-pMCS5	GATATTGTTCAGCGCGGC	
63.	HR3000-fr-pMCS5	TACCGATGCGATGGCCTAC	Primers used amplify HR3000 fragment with BW25113

64.	HR3000-rev-pMCS5	TCAAGTTCCTCTGCTGTAAGT	genome as template, which was used to construst pBBR1MCS5-Spc::HR3000
65.	pCL-fr-Ntac	TATGGTGCACTCTCAGTACAATCT	Primers used amplify linearized vector with pCL1920 as template, which was used for pCL1920::Ntac-eGFP cloning with 20bp homologous ends
66.	pCL-rev-Ntac	TCCAGCAAAGGTCTAGCAGAA	
67.	Ntac-fr-pCL	TCTGCTAGACCTTTGCTGGA	Primers used amplify Ntac fragment with pNTG as template, which was used for pCL1920::Ntac-eGFP cloning with 20bp homologous ends
68.	Ntac-fr-pCL	TGTACTGAGAGTGCACCATATGC	
69.	pCL-fr-2TR	GAATCGTTTTCCGGGACGC	Primers used amplify linearized vector with pCL1920::pkat-eGFP as template, which was used to construct pCL1920::2TR-300bp, pCL1920::2TR-1000bp
70.	pCL-rev-2TR	CGAAGCCCAACCTTTCATAGAAG	
71.	2TR-300-fr-pCL	GGGAACTATAAGACACGTGCTACATCATGGCAGACAAACAAAAG	Primers used amplify TR300 fragment with pCL1920::pkat-eGFP as template, which was used to construct pCL1920::2TR-300bp
72.	2TR-300-rev-pCL	TCTATTAACAAGTGTATCACTTGGTCGGTCATTTCGAACCCCAGA	
73.	pCL-fr-3TR-300	GTGATACACTTGTTAATAGAATCGAG	Primers used amplify linearized vector with pCL1920::2TR-300bp as template, which was used to construct pCL1920::3TR-300bp
74.	pCL-rev-3TR-300	GCACGTGTCTTATAGTTCCCGT	
75.	3TR-300-fr-pCL	GGGAACTATAAGACACGTGCTACATCATGGCAGACAAACAAAAG	Primers used amplify TR300 fragment with pCL1920::pkat-eGFP as template, which was used to construct pCL1920::3TR-300bp
76.	3TR-300-rev-pCL	TCTATTAACAAGTGTATCACTTGGTCGGTCATTTCGAACCCCAGA	

77.	2TR-1000-fr-pCL	CCGTGACAGGTCATTCAGACGGGACTGGGCTATCTGGACAAG	Primers used amplify TR1000 fragment with pCL1920::pkat-eGFP as template, which was used to construct pCL1920::2TR-1000bp
78.	2TR-1000-rev-pCL	CTTACTGGGTGCATTAGCCATTGGTCGGTCATTTCGAACCCCAGA	
79.	pCL-fr-3TR-1000	TGGCTAATGCACCCAGTAAGGC	Primers used amplify linearized vector with pCL1920::2TR-1000bp as template, which was used to construct pCL1920::3TR-1000bp
80.	pCL-rev-3TR-1000	GTCTGAATGACCTGTCACGGGATA	
81.	3TR-1000-fr-pCL	CCGTGACAGGTCATTCAGACGGGACTGGGCTATCTGGACAAG	Primers used amplify TR1000 fragment with pCL1920::pkat-eGFP as template, which was used to construct pCL1920::3TR-1000bp
82.	3TR-1000-rev-pCL	CTTACTGGGTGCATTAGCCATTGGTCGGTCATTTCGAACCCCAGA	
83.	pCL-fr-NTR	GTCTGCTATGTGGTGCTATCTG	Primers used amplify linearized vector with pCL1920 as template, which was used for pCL1920::NTR-300bp and pCL1920::NTR-1000bp cloning with 20bp homologous ends
84.	pCL-rev-NTR	GCCTCTTCGCTATTACGCCA	
85.	NTR-fr-pCL	TGGCGTAATAGCGAAGAGGC	Primers used amplify NTR fragment with pCL1920::NTR-300bp and pCL1920::NTR-1000bp as template, which was used for pCL1920::NTR-300bp and pCL1920::NTR-1000bp cloning with 20bp homologous ends
86.	NTR-rev-pCL	CAGATAGCACCACATAGCAGAC	

Supplementary Table S3. Positive rates of homologous inserts into four plasmids with BW25113, BWΔrecA and XL1-Blue MRF'

Plasmids		Positive rates		
		**BW25113** (%)	**BWΔrecA** (%)	**XL1-Blue MRF'** (%)
Vector :: NKan-Gm-CKan-300bp (Fig. 2A)	pSK::NKan-Gm-CKan-300bp	97 ± 1	94 ± 0	97 ± 1
	pCL1920::NKan-Gm-CKan-300bp	97 ± 0	97 ± 0	94 ± 2
	pBR322::NKan-Gm-CKan-300bp	97 ± 0	92 ± 1	93 ± 1
	pMCS5::NKan-Gm-CKan-300bp	97 ± 0	91 ± 1	92 ± 1
Vector :: HR3000 (Fig. 2B)	pSK::HR3000	95 ± 1	95 ± 1	95 ± 0
	pCL1920::HR3000	95 ± 1	100 ± 0	100 ± 0
	pBR322::HR3000	100 ± 0	95 ± 1	95 ± 1
	pMCS5::HR3000	90 ± 0	90 ± 1	100 ± 0

[a] The data are averages of clone colonies from three tests (with standard deviations).

Supplementary Table S4. The plasmids extracted in the plasmid stability assay

Plasmids	Strains	Colonies [a]	Concentration of plasmids before 3 transfers (ng/ul) [b]	Concentration of plasmids after 3 transfers (ng/ul) [b]
pSK::NKan-Gm-CKan-300bp	BW25113	R1	514.7	520.67
	BW25113	R2	606.4	492.6
	BWΔrecA	PC	357.13	312.51
pCL1920::NKan-Gm-CKan-300bp	BW25113	R1	26.5	14.85
	BW25113	R2	18.9	22.829
	BWΔrecA	PC	27.74	38.76
pBR322::NKan-Gm-CKan-300bp	BW25113	R1	69.937	67.43
	BW25113	R2	52.518	50.7
	BWΔrecA	PC	73.52	55.56
pMCS5::NKan-Gm-CKan-300bp	BW25113	R1	24.49	29
	BW25113	R2	43.05	43.015
	BWΔrecA	PC	29.06	31.05
pSK::HR3000	BW25113	R1	236.7	44.8
	BW25113	R2	372.68	227.49
	BWΔrecA	PC	500	416.67
pCL1920::HR3000	BW25113	R1	38.222	23.33
	BW25113	R2	32.523	47.552
	BWΔrecA	PC	54.34	36.23
pBR322::HR3000	BW25113	R1	51.029	51.76
	BW25113	R2	31.909	28.8
	BWΔrecA	PC	102.04	89.90
pMCS5::HR3000	BW25113	R1	32.562	31.24
	BW25113	R2	44.176	31.86
	BWΔrecA	PC	36.76	40.01
pCL1920::8tac-eGFP	BW25113	R1	30.864	22.13
	BW25113	R2	17.123	16.39
	BWΔrecA	PC	13.298	17.67
pCL1920::3TR-300	BW25113	R1	34.72	84.75
	BW25113	R2	42.02	44.64
	BWΔrecA	PC	17.79	29.94
pCL1920::3TR-1000	BW25113	R1	22.94	17.18
	BW25113	R2	25	15.52
	BWΔrecA	PC	18,31	26.73

[a] R1 and R2 means the plasmids were extracted from two clonies of BW25113, and PC means the plasmid was extracted from BWΔrecA used as positive control.

[b] The plasmids were extracted from 3 mL of overnight cultures and dissolved in 45 ul of H_2O.

REFERENCES

Xia, Y.Z., Li, K., Li, J.J., Wang, T.Q., Gu, L.C., and Xun, L.Y. (2019). T5 exonuclease-dependent assembly offers a low-cost method for efficient cloning and site-directed mutagenesis. *Nucleic Acids Research* 47(3)**,** e15. doi: 10.1093/nar/gky1169.
